# Assessing Perceived Need for Mental Healthcare Among Adults in Germany

**DOI:** 10.3389/ijph.2025.1607927

**Published:** 2025-06-03

**Authors:** Lena Walther, Felicitas Vogelsang, Julia Thom, Heike Hölling, Thomas G. Grobe, Timm Frerk, Ursula Marschall, Diana Peitz

**Affiliations:** ^1^ Department of Epidemiology and Health Monitoring, Robert Koch Institute, Berlin, Germany; ^2^ Department of Health Monitoring and Biometrics, aQua-Institute, Göttingen, Germany; ^3^ Institut für Gesundheitssystemforschung, BARMER Krankenkasse, Wuppertal, Germany

**Keywords:** perceived need, mental healthcare, public mental health, mental health surveillance, patient perspective

## Abstract

**Objectives:**

To describe the prevalence and distribution of perceived need for mental healthcare among adults in Germany and examine its association with more objective indicators of need as well as mental health literacy.

**Methods:**

We used data from 6,558 adults randomly sampled from a health insurance company as well as nationally representative survey data from 10,676 adults. Prevalence estimates were calculated, also by sex, age and education. Bivariate and multivariate associations between perceived need and sociodemographic characteristics, psychopathological symptoms, functional impairment and F-diagnoses as well as mental health literacy were examined.

**Results:**

Approximately one-sixth of adults perceived a need for mental healthcare in the previous 12 months. Perceived need was associated with female sex (bivariate association only), younger age, high educational attainment, psychopathological symptoms, mental health-related functional impairment and mental health literacy. Those with perceived need were also twice as likely to have a documented F-diagnosis than those without.

**Conclusion:**

Perceived need should be monitored within mental health surveillance to inform healthcare planning from a patient perspective and address the mental health treatment gap.

## Introduction

Mental health is an essential part of overall population health. Mental disorders, defined as psychopathological syndromes accompanied by significant distress and functional impairment [1], are among the leading contributors to the burden of disease worldwide [2, 3]. The provision of accessible and adequate mental healthcare should therefore be a public health priority. However, assessing and monitoring need for mental healthcare in populations for the purposes of evidence-based healthcare system planning is not straightforward [4–6].

Different measures indicating how widespread mental disorders are at a given time and how the mental health status of a population has developed represent a major source of information on the possible magnitude of mental healthcare need [7]. One such measure is the prevalence of mental disorder diagnoses captured in routine data from the healthcare system, i.e., the proportion of those participating in a healthcare system who received a mental disorder diagnosis within a certain time period. Data on diagnoses documented by health professionals uniquely captures “normative need,” defined as having been identified by a health practitioner [8]. It also has the advantage of showing developments in the real-world clinical settings that exist to meet mental healthcare needs. However, this data only includes cases that have come to the attention of a care provider and is further limited by the fact that practitioner diagnoses may not always reflect morbidity accurately [9–11]. Another key source of information on possible treatment needs is survey data on the prevalence of mental disorders in the general population assessed using psychodiagnostic interviews (e.g., [12]) and on symptoms of mental ill health as well as mental health-related functional impairment assessed using brief self-report instruments. Symptoms captured in these epidemiological studies might be transient or mild, however, meaning that specialised treatment may not be required in all cases [13]. Critically, the presence of mental disorder symptoms has been found to be an inadequate predictor of treatment seeking, contributing to the so called “treatment gap” in mental healthcare [12, 14–16]. These limitations suggest that additional measures may be beneficial.

A separate, subjective dimension of need has been found to be crucial in explaining the mental health treatment gap [17, 18] and in predicting whether an individual actually seeks professional help or not [19]: The patient-centred indicator “perceived need for mental healthcare” [20] or “felt need” [8] reflects the extent to which individuals themselves are aware of having mental health problems and believe that their problems necessitate professional help [21]. A large-scale European study found those with a disabling 12-month mental disorder who perceived a need for treatment were eight times more likely to be in care than those with a disabling disorder but without perceived need [18]. Perceived need is thought to be intricately linked to mental health literacy (MHL) [22], which encompasses mental health knowledge and personal attitudes towards mental disorders and their treatment [19, 23, 24]. Related to overall deficits in MHL in populations, fear of stigmatisation is also among the factors that can prevent individuals from identifying and expressing need for professional care [21, 25]. Perceived need was recognised as an important predictor in Andersen and Davidson’s influential behavioural model of healthcare service use [20]. It has been found to be strongly associated with mental ill health [13, 19, 26, 27] as well as poor quality of life and suicidal ideation independent of whether criteria for common mental disorders as defined in the Diagnostic and Statistical Manual of Mental Disorders (DSM) [28] are met [13].

Because of its link to help-seeking behavior, overall mental health, and also in light of increasing recognition of the importance of the patient perspective within public mental health [27, 29], perceived need may be an important additional measure to monitor in populations for evidence-based healthcare planning. Therefore, perceived need is a potential indicator to be observed as part of the surveillance of mental health in Germany [30], following the example of e.g., Australia [27]. The candidate operationalisation is an economical single survey item which has not been previously employed in Germany.

Overall, data on the prevalence of perceived need in the German population is scarce: We found only one study presenting data on perceived need for two separate time periods using a different instrument, which showed that 11.8% of adults in Germany reported having had an emotional problem that required medical or psychological help in the previous 3 years in 2014 and 14.0% in 2019 [21, 31]. Furthermore, the association between perceived need and the other measures of need addressed above has not, to our knowledge, been explored in Germany’s population. A better understanding of the meaning of perceived need reports using the candidate single item is required as a basis for future surveillance: Perceived need can more readily be incorporated as a surveillance indicator informing health service planning if it is linked to psychopathology and functional impairment, given that they represent the intended basis for clinician diagnosis of a mental disorder (normative need) and, in turn, the allotment of resources in the healthcare system. Meaningful triangulation between this subjective measure of need and the more objective measures described above (symptoms, impairments and diagnoses) as a way of addressing the different measures’ limitations also requires that they are linked and do not reflect completely separate phenomena (see definition of public health triangulation as “reviewing, synthesising and interpreting [secondary] data from multiple sources that *bear on the same question*” [32]).

To address these research gaps, the present work uses data from two surveys and data linkage to insurance claims to 1) describe the prevalence of perceived need among adults in Germany, including its distribution by gender, age and education level. We also 2) examine whether perceived need is associated with psychopathological symptoms and functional impairment (commonly used as a basis for deciding on normative need) as well as the actual diagnosis of a mental health disorder by a health professional. For a better understanding of perceived need, we 3) additionally examine the association between perceived need and MHL given its potential relevance to people’s ability to recognise and report need, as outlined above.

## Methods

### Procedure and Participants

We used data from two separate studies to provide more robust evidence.

Study 1 is the German data linkage project *Optimised Data for Public Mental Health*, methods described elsewhere [33]. It comprises survey data (paper and pencil interviewing) collected between October and December 2021 linked on the person level to insurance claims data from *n* = 6,558 participants randomly sampled among adults insured with a major German statutory health insurance company (BARMER).

We repeated some analyses with data from the representative telephone survey *German Health Update* (GEDA, Study 2), methods described elsewhere [34]. We used data from *n* = 10,676 randomly sampled participants from the adult population living in Germany interviewed between July 2021 and October 2022.

To counteract possible biases due to selective participation, adjustment weighting by population distributions of gender, age, region (2020 statistics from the Federal Statistical Office) and education (Microcensus 2018) was applied to both datasets across analyses.

Sample characteristics are summarised in Table 1. We defined five age groups (18–29, 30–44, 45–64, 65–79 and 80+ years). The CASMIN Educational Classification (Comparative Analyses of Social Mobility in Industrial Nations) was used to categorise participants’ level of education into low, middle and high based on self-reported information on school-leaving and post-school qualifications [35].

**TABLE 1:** Sample characteristics (Study 1: Optimised Data for Public Mental Health, Study 2: German Health Update, Germany, 2021-2022).

Characteristic	Category	Study 1		Study 2	
		Weighted %	Unweighted n	Weighted %	Unweighted n
Total (N)		100%	6,558	100%	10,676
Sex (at birth)	Female	51.1%	3,686	51.1%	5,684
	Male	48.9%	2,872	48.9%	4,992
Age group	18–29 years	16.0%	657	16.1%	833
	30–44 years	22.9%	1,032	22.6%	1,687
	45–64 years	34.8%	2,409	35.2%	4,146
	65–79 years	17.8%	1,713	17.6%	2,863
	80+ years	8.6%	747	8.6%	1,147
Level of education^a^	Low	26.0%	1,509	26.5%	1,788
	Middle	57.0%	3,498	53.7%	4,605
	High	17.0%	1,497	19.8%	4,244

Note.

^a^ In accordance with the CASMIN classification system (see *Methods* section).

### Measures

#### Perceived Need for Mental Healthcare

We used a single-item measure from the United States’ National Comorbidity Survey [19, 26] and the World Mental Health (WMH) surveys [36] to capture *perceived need for mental healthcare* in the past 12 months: “*Was there ever a time during the past 12* *months when you felt that you might need to see a professional because of problems with your emotions or nerves or your use of alcohol or drugs?*” (“*Yes*” or “*No*”). The item was translated into German using back translation. German language particularities meant that the equivalent of “to seek professional help” was preferable to “to see a professional.”

#### Psychopathology

To screen for psychopathological symptoms, we used the Patient Health Questionnaire-2 (PHQ-2 [37]) and the Generalised Anxiety Disorder Scale-2 (GAD-2 [38]), measures of two core depressive/anxiety symptoms (cutoffs PHQ-2 > 2 and GAD-2 > 2, responses *0 = “not at all” *to* 3 = “nearly every day”*), the Patient Health Questionnaire-Panic Disorder (PHQ-PD, four-item version [39, 40]; measure of panic symptoms with affirmative responses on all four questions indicating a positive screen; binary responses*, “yes,” “no”*) and Primary Care PTSD Screen for DSM-5 (PC-PTSD-5 [41, 42]; measure of post-traumatic stress symptoms with affirmative responses on first question and at least three of the five remaining questions indicating a positive screen). The PC-PTSD-5 was only included in Study 1. Additionally, the binary variable “positive on any screener” (yes/no) was constructed using the PHQ-2, GAD-2, PHQ-PD and PC-PTSD-5 for Study 1 (“yes” in case of positive screen on at least one of these instruments) and PHQ-2, GAD-2 and PHQ-PD for Study 2.

As a measure indicating psychopathology as identified by healthcare practitioners (“normative need”), F-diagnoses (International Statistical Classification of Diseases and Related Health Problems (ICD-10) [43] codes F00-F99) documented in insurance claims (Study 1) in the previous 12 months (4th quarter 2020 to 3rd quarter 2021) were examined. Additionally, diagnoses which were prioritised for mental health surveillance [30] or very frequent in ambulatory care [44] were examined individually: depressive disorders and dysthymia (F32, F33, F34.1; referred to as “depression” below), phobic and anxiety disorders (F40, F41; “anxiety”), post-traumatic stress disorder (PTSD, F43.1), schizophrenia, schizotypal and delusional disorders (F2; “schizophrenia”), somatoform disorder (F45) and adjustment disorder (F43.2).

#### Functional Impairment

We used the mental component score (MCS) as well as the physical component score (PCS) of the Short-Form Health Survey (SF-12 [45]; point range: 0–100; data from Study 1) as metric measures of health-related quality of life to assess functional impairment. Lower scores indicate greater functional impairment.

#### Mental Health Literacy

The total score of the Mental Health Knowledge Schedule (MAKS [46]; point range: 12–60; data from Study 1) was used to assess stigma-related mental health knowledge, and the total score of four items from the Mental Health Literacy Scale was used to capture further attitudes toward mental illness (“A mental illness is not a real medical illness,” “People with a mental illness could just snap out of it if they wanted,” “If I had a mental illness I would not tell anyone,” “If I had a mental illness, I would not seek help from a mental health professional”) (MHLS [47]; point range: 4–20; data from Study 1).

### Statistical Analyses

We calculated total percentages with 95% confidence intervals of those who reported a perceived need for mental healthcare in Studies 1 and 2 as well as percentages within sex, age and level of education subgroups in order to assess the prevalence of perceived need and its distribution in the population (aim 1). To examine whether perceived need is associated with psychopathological symptoms, functional impairment and mental disorder diagnoses, we compared these indicators of psychopathology in those with and without perceived need (aim 2). Specifically, we calculated a) percentages of those above the cutoff scores on each of the specific screeners named above as well as any psychopathology screener for each group (Study 1 & 2), b) percentages of individuals with any documented F-diagnoses as well as with each of the selected F-diagnoses for both groups (Study 1) and c) mean functional impairment scores for both groups. To additionally examine the association between perceived need and MHL (aim 3), we also calculated mean MHL by perceived need status. Chi-squared tests and t-tests were used for statistical comparisons for categorical and metric variables, respectively.

Using data from Study 1, we also calculated a multivariate logistic regression model in order to examine the association between perceived need and sociodemographic characteristics, measures of mental health (positive on any screener), functional impairment and MHL whilst controlling for each of the respective other variables. This analysis targets all three study aims, providing an improved understanding of the distribution of perceived need by sociodemographic group (aim 1) as well as of the association between perceived need and more objective measures of need (aim 2) and MHL (aim 3) by elucidating whether observed patterns are explained by the respective other factors. Age was included in all analyses as a categorical variable owing to the relevance of information on particular age groups within public health surveillance as an evidentiary basis for policy as well as to potential non-linear relationships between age and the other variables examined. Due to their unique relationship to the outcome variable – with diagnoses often being a result of care seeking and care seeking often indicating perceived need, we did not include documented diagnoses in this analysis. We tested for multicollinearity among the predictor variables and found a variance inflation factor (VIF) below 2 across variables. Because all scales used comprised a limited range of values, no extra analyses for outliers were necessary. We also checked the dataset for implausible values. All analyses using data from Study 1 were computed with SAS 9.4 [48], while all analyses using data from Study 2 were computed with STATA 17 [49].

## Results

Table 2 shows estimates of percentages with perceived need for mental healthcare for the total samples as well as for subgroups by sex, age and education.

**TABLE 2:** Perceived need for mental healthcare by sociodemographic characteristics (Study 1: Optimised Data for Public Mental Health, Study 2: German Health Update, Germany, 2021-2022).

Characteristic	Category	Perceived need	
		Study 1	Study 2
Total		17.9% [16.7–19.9]	15.8% [14.7–16.9]
Sex (at birth)	Female	21.2% [19.7–22.8]	19.2% [17.6–20.1]
	Male	14.4% [12.6–16.1]	12.2% [10.7–13.8]
Age group	18–29 years	31.6% [27.4–35.8]	26.9% [23.0–31.1]
	30–44 years	23.2% [20.2–26.1]	20.5% [17.9–23.4]
	45–64 years	17.0% [15.3–18.6]	15.8% [14.1–17.6]
	65–79 years	7.1% [5.7–8.5]	5.7% [4.7–7.0]
	80+ years	3.7% [2.2–5.2]	3.0% [1.9–4.5]
Level of education^a^	Low	11.5% [9.3–13.7]	12.2% [10.1–14.7]
	Middle	19.9% [18.2–21.5]	16.9% [15.3–18.6]
	High	20.3% [18.0–22.6]	17.7% [16.0–19.6]

Note.

^a^ In accordance with CASMIN. Weighted data. 95% confidence intervals shown in brackets. All differences between population subgroups shown by non-overlapping confidence intervals were significant at *p* < 0.0001 according to Chi-squared tests.

In both datasets, approximately one-sixth of participants (Study 1: 18%; Study 2: 16%) reported a perceived need for mental healthcare. A greater proportion of females than males reported perceived need in both studies (Study 1: 21% vs. 14%; Study 2: 19% vs. 12%). The likelihood of reporting need appears to decline with age, with approximately 32% (27% in Study 2) of those aged 18–29 years responding in the affirmative compared to 17% (16% in Study 2) among those aged 45–64 and just 4% among those aged 80+ (3% in Study 2). Stratification by educational attainment showed less reported need for the low level of education group (12% in Study 1 and 2) compared to the middle and high level of education groups (both between 17% and 20% in Study 1 and Study 2).

Table 3 shows results on psychopathological symptoms, mental disorder diagnoses and functional impairment as well as MHL in participants with and without a perceived need for mental healthcare in Studies 1 and 2. We find that those who reported perceived need were far more likely to have scored above cutoff on all screening instruments for psychopathology than those who did not. 63% (Study 2: 47%) of those with perceived need scored above cutoff on any of these screeners. Insurance claims data from Study 1 further revealed a higher percentage with a documented F-diagnosis in the previous 12 months among those who reported a need for mental healthcare for this time period than in those who did not (61% vs. 34%). The same was true for each individual diagnosis examined (depression, anxiety, PTSD, schizophrenia, somatoform disorders and adjustment disorders). Individuals reporting need also showed far lower levels of mental as well as slightly lower levels of physical health-related quality of life, i.e. greater functional impairment (Study 1).

**TABLE 3:** Psychopathological symptoms, mental disorder diagnoses, functional impairment and mental health literacy among participants with and without a perceived need for mental healthcare. (Study 1: Optimised Data for Public Mental Health, Study 2: German Health Update, Germany, 2021-2022).

Outcome	Data source	Perceived need	
		Yes	No
**Psychopathological symptoms – percentage affected with 95% CIs**			
Depressive symptoms (PHQ-2)	Study 1	42.6% [38.9–46.2]	7.6% [6.7–8.5]
	Study 2	35.9% [32.1–39.9]	11.7% [10.6–12.8]
Anxiety symptoms (GAD-2)	Study 1	44.9% [41.2–48.5]	6.4% [5.5–7.2]
	Study 2	31.7% [28.1–35.6]	4.5% [3.8–5.2]
Panic symptoms (PHQ-PD)	Study 1	13.4% [10.9–16.0]	1.3% [1.0–1.7]
	Study 2	12.6% [10.1–15.6]	0.9% [0.6–1.3]
PTSD symptoms (PC-PTSD-5)	Study 1	19.7% [16.7–22.8]	2.6% [2.1–3.1]
Positive on any screener	Study 1	62.9% [59.4–66.4]	15.3% [14.1–16.5]
	Study 2	46.6% [42.4–50.3]	9.5% [8.5–10.5]
**Mental disorder diagnoses – percentage with diagnosis with 95% CIs**			
Any F-Diagnosis (F00-99)	Study 1	61.0% [57.4–64.7]	33.7% [32.1–35.2]
Depression (F32, F33, F34.1)	Study 1	37.9% [34.4–41.4]	11.6% [10.7–12.6]
Anxiety (F40, F41)	Study 1	18.2% [15.3–21.1]	4.8% [4.1–5.5]
PTSD (F43.1)	Study 1	3.0% [1.9–4.2]	0.5% [0.2–0.7]
Schizophrenia, schizotypal and delusional disorders (F2)	Study 1	1.2% [0.4–2.0]	0.4% [0.1–0.6]
Somatoform disorder (F45)	Study 1	17.3% [14.8–19.8]	10.1% [9.1–11.0]
Adjustment disorder (F43.2)	Study 1	11.7% [9.4–14.0]	3.0% [2.4–3.6]
**Functional impairment – means with 95% CIs**			
Mental health-related quality of life (MCS of SF-12)^a^	Study 1	36.8 [36.0–37.6]	50.6 [50.3–50.9]
Physical health-related quality of life (PCS of SF-12)^a^	Study 1	50.5 [49.8–51.2]	48.7 [48.4–49.0]
**Mental health literacy – means with 95% CIs**			
Mental health-related knowledge (MAKS)	Study 1	44.9 [44.5–45.4]	43.6 [43.4–43.8]
Attitudes toward mental illness (4 items from MHLS)	Study 1	22.2 [22.0–22.4]	21.5 [21.4–21.6]

Note. Weighted data. “CIs” = “confidence intervals”. All differences between those with and without perceived need were significant (*p* < 0.0001) according to Chi-squared tests and t-tests (only p-value for F2 diagnoses is larger; p = 0.0041). The variable “positive on any screener” includes the PHQ-2, GAD-2, PHQ-PD and PC-PTSD-5 for Study 1, and PHQ-2, GAD-2, and PHQ-PD for Study 2.

^a^ With regard to the health-related quality of life indicators, lower scores indicate higher functional impairment.

MHL scores were significantly higher for those with perceived need for mental healthcare along both measures (mental health-related knowledge and attitudes toward mental illness); however, differences in mean scores were small. Further analyses showed that those who did not report need but screened positive on at least one screener (15% of participants in Study 1) also had significantly lower MHL than those who screened positive and perceived a need for help [mean MAKS score: 42.0 (41.4–42.5) vs. 44.5 (43.9–45.1); MHLS: 14.5 (14.2–14.8) vs. 15.9 (15.6–16.2)].

**FIGURE 1:**
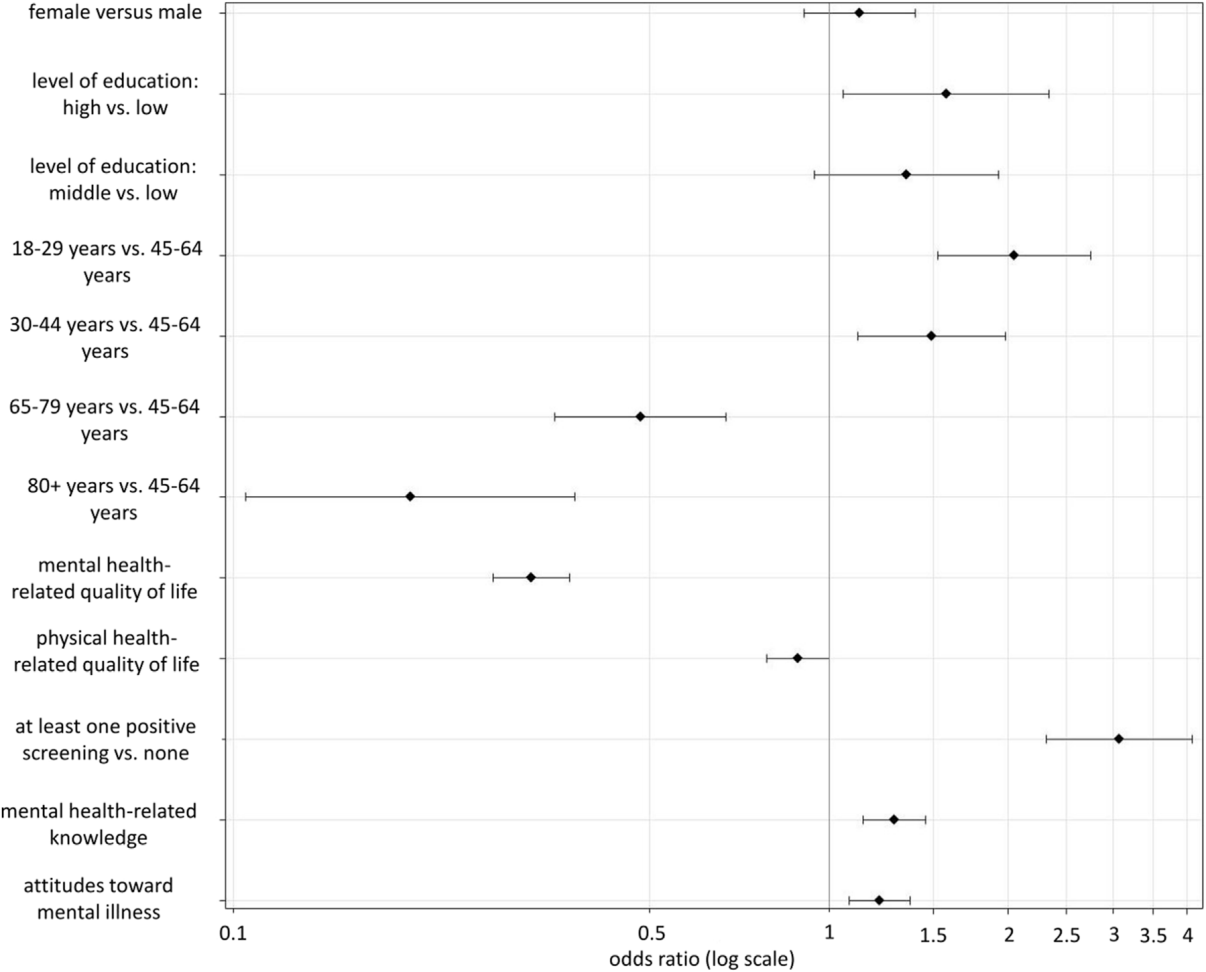
Logistic regression predicting perceived need of mental healthcare (Germany, 2021). Note. Weighted data from Study 1: Optimised Data for Public Mental Health. Error bars show 95% confidence intervals for the odds ratios. With regard to the health-related quality of life indicators, lower scores indicate higher functional impairment.

Results from the multivariate analysis (Figure 1, data from Study 1) confirmed almost all of the above findings and show that age is a major predictor of perceived need, with higher age (65+ years) associated with lower odds of perceived need and lower age (<45 years) associated with greater odds. A positive screen on any of the employed psychopathology screeners is also a strong predictor of perceived need, and mental health-related functional impairment shows a positive relationship with perceived need (i.e., lower MCS scores predict perceived need). Controlling for all other characteristics, sex and physical health-related quality of life are no longer significantly associated with perceived need, while high but not middle level of education remains predictive of perceived need compared to low level of education. Multivariate analysis also shows a significant positive association between MHL and perceived need.

## Discussion

The present study aimed to 1) describe the prevalence of perceived need for mental healthcare among Germany’s adult population and its distribution among population subgroups, 2) examine whether perceived need measured using a single survey item is associated with psychopathological symptoms, functional impairment and documented F-diagnoses with the larger aim of assessing its suitability for surveillance and 3) further improve our understanding of perceived need as an indicator by examining its association with MHL.

### Perceived Need in the Population

One-sixth of the population reported a perceived need for mental healthcare in the previous 12 months in 2021/2022 (Study 1: 18%, Study 2: 16%). This figure is remarkably consistent with findings from other high-income countries from (over) a decade ago (Australia: 14% with perceived need in 2007 [27]; Canada: 17% with perceived need in 2012 [50]) as well as a previous 3-year prevalence estimate for Germany (14%) based on 2019 survey data [31]. One-sixth of adults with perceived mental healthcare needs in the previous year stands in contrast to a 12-month mental disorder prevalence of approximately one-third among adults in Germany as reported based on clinical interview data from 2009–2012 [12].

In line with previous studies, perceived need for mental healthcare was reported more frequently by females [31, 51], younger adults [52, 53] and those with higher educational attainment [26, 53]. The gender gap in perceived need is consistent with higher prevalence of mental disorders among women [12] as well as higher treatment seeking and mental healthcare use among women than men in Germany [15, 54–56]. Notably, the association between sex and perceived need was not statistically significant in the multivariate analysis, suggesting that the bivariate relationship is explained by other related factors such as higher symptom burden, higher functional impairment or differences in MHL (found in our data [results not shown] and in a previous German study [57]), rather than by a more immediate gender role effect on the expression of needs.

Age group differences in perceived need also match age group differences in psychopathology, 2009–2012 survey estimates showing a declining prevalence of mental disorders with age [12]. Consistent with our findings of particularly low levels of perceived need among older adults, the oldest adults with a mental disorder were least likely to report contact to mental healthcare services according to the same data [15]. Higher perceived need among the youngest adults, however, may not always translate into increased treatment seeking: The same clinical interview study did not find increased self-reported service use among younger compared to middle-aged adults affected by a mental disorder [15]. A nationally representative survey asking generally about mental healthcare use in the previous year reported peak use in middle age [56]. Barriers to treatment seeking among young adults with perceived need and barriers to perceiving need among older adults therefore warrant attention. Importantly, unlike sex, age remained a strong predictor of perceived need even after controlling for psychopathology, functional impairment and MHL (which is higher among younger adults, results not shown), suggesting that other age-specific factors explain the observed differences. Long-term surveillance should aim to disentangle age effects from possible cohort effects on the prevalence of perceived need for care [58].

In contrast to patterns observed by sex and age, prevalence of psychopathology and perceived need come apart in the educational attainment groups. While individuals with a low level of education are more frequently affected by mental health problems [59], this group is less likely to report need for mental healthcare than those with a high level of education, with and without control for symptoms, functional impairment and even MHL – which is a strong candidate factor in explaining this discrepancy [26, 60]. This result is in line with findings of equal self-reported service use despite differences in psychopathology by education [15] and socioeconomic status [56] in Germany, as well as national studies showing lower utilisation rates of psychotherapy among those with low levels of education according to self-reports [31] and registry data from statutory health insurance [61]. It has been proposed that disadvantaged groups may avoid mental healthcare due to a greater fear of stigmatisation, lack of trust in mental health professionals as well as a lack of resources negatively impacting healthcare access [60], which is a potential barrier even when treatment itself is covered by insurance. Lower socioeconomic status individuals may also be more likely to attribute their symptoms to their life circumstances rather than to a mental disorder [26]. This underscores the importance of efforts aimed at reducing social inequality in health [59, 62] alongside improving equality in healthcare access, which includes needs awareness.

### Association Between Perceived Need and Psychopathological Symptoms, Functional Impairment and Documented Diagnoses

The relevance of perceived need as a mental health surveillance indicator used to inform evidence-based healthcare planning depends on its association with measures of need that are meant to determine the allotment of healthcare resources – namely, indicators capturing psychopathology and functional impairment, which are also the intended basis for clinician diagnosis of a mental disorder. Its association with these other measures is also a prerequisite for meaningful triangulation between needs indicators within surveillance. Indeed, we find strong associations between perceived need and psychopathological symptom burden (4-5 times as many above-cutoff scores on any screener among those reporting need compared to those not reporting need) as well as an independent association with mental health-related functional impairment, consistent with the literature [6, 13]. The fact that by far not all individuals reporting a need for care screened positive for psychopathological symptoms is to be expected given the limited scope of the screening instruments as well as the differing temporal reference periods of these instruments (2/4 weeks) and the perceived need item (12 months).

Our finding of a nearly twofold prevalence of documented F-diagnoses (F00-99) in the previous 12 months in the group with perceived need compared to the group without and between two to six times the individual diagnoses examined sheds light on the relationship between perceived need and “normative need” [8]: 61% of those with perceived need have had need identified by a health professional. Of course, these results are also bound to be heavily impacted by the close relationship between perceived need and help seeking. Notably, 33% of those without a perceived need for treatment in the previous 12 months have a documented F-diagnosis in this period. This may be because F-diagnoses cover a broad scope and not all diagnoses may be perceived as “problems with emotions, nerves or use of alcohol or drugs.” Generally, perceived need likely varies by disorder just as treatment seeking does [15]. This means that even among those who have had contact to health services that results in diagnosis, identification and reporting of need is likely to vary. However, we find a non-negligible proportion with a documented diagnosis among those without perceived need for each of the specific diagnosis groups examined. In line with the pursuit of patient-centred approaches to mental healthcare [27] and given potential for improvements in diagnostic practice [10, 11], this mismatch should be addressed in healthcare practice.

### Association Between Perceived Need and MHL

While it bears repeating that MHL does not (fully) explain the fact that those with a low level of education are less likely to report need but more likely to have mental health problems, an association between MHL and perceived need was found. Furthermore, those with symptoms but no perceived need exhibited significantly lower MHL than those who had symptoms and reported need. These findings are in support of the idea of improving MHL as an approach for improving access to care and reducing the treatment gap. However, MHL score differences between those with and without perceived need were surprisingly small, suggesting that future research could benefit from exploring whether other measures of MHL reveal more variance, particularly as MHL is likely ever evolving in populations.

### Limitations

The following limitations should be considered: 1) The comparability between Study 1 and Study 2 is limited by differences in survey mode (written self-report versus telephone interview) as well as how representative of the population of adults living in Germany the data can be assumed to be (random sampling from members of an insurance company comprising over 10% of the German population versus from the general population). However, differences between the insurance sample and the German population according to age, gender, region and education are minimised using weighting factors. Further differences in certain combinations of age, gender, region and education in the sample are assumed not to have a large impact on the overall results [63]. A strength of our study is that we were able to use a sample randomly drawn from the general population to reproduce some analyses from Study 1. The similarity in results from the two studies can be seen as evidence for the representativeness of the results based on insuree data. 2) While the employed single-item measure of perceived need has been used in several large-scale studies internationally, it has not been validated using longer perceived need assessments such as the one used in Meadows and colleagues [27] or qualitative assessments of item interpretation. 3) The employed screeners cover a limited range of symptoms and do no refer to the same time period as the perceived need measure, limiting the extent to which outcomes can be used to assess the meaning of perceived need. 4) All self-report measures may be subject to social desirability and recall bias effect.

### Conclusion

The present study contributes to a better understanding of a single-item measure of perceived need and provides evidence for its suitability as a measure to be monitored within public health surveillance. The integration of different indicators including prevalence of mental disorders, symptoms and functional impairment in the population, prevalence of F-diagnoses in the healthcare system as well as the patient-centred indicator perceived need may be the best approach to the difficult task of evidence-based healthcare planning, as has been previously argued [6, 13].

Research should continue to examine possible factors explaining the absence of perceived treatment need among those with mental health problems with the aim of informing public health measures seeking to improve access to care. These measures should be conceived with the distribution of perceived need within different sociodemographic groups, particularly by age and education, in mind. Improving MHL in the population may be one approach to reducing the treatment gap, but our finding that perceived need differences by age and education persist after controlling for MHL suggest that this may not be sufficient. Another approach is to circumvent perceived need and introduce symptom screening in general healthcare practice, particularly among high-risk groups [64, 65]. However, efforts to overcome the mental health treatment gap should not undermine the patient perspective on need for care. For various reasons, professional care may not always be the desired or most impactful approach to addressing symptoms of mental ill health – particularly with regard to the social determinants of mental health [59]. Future research should examine met versus unmet perceived need and factors linked to unmet perceived need in Germany.
